# Viral infections alter the human salivary volatilome

**DOI:** 10.1128/msphere.00222-26

**Published:** 2026-05-11

**Authors:** Bruce A. Kimball, Gary K. Beauchamp, Olivia F. Taylor, Susan E. Coffin, Frances B. Balamuth, Juliana Jones, Audrey R. John, Virginia A. Stallings

**Affiliations:** 1 Monell Chemical Senses Center 10630, Philadelphia, Pennsylvania, USA; 2 Children’s Hospital of Philadelphia, Philadelphia, Pennsylvania, USA; 3 Department of Pediatrics, Perelman School of Medicine, University of Pennsylvania 6572, Philadelphia, Pennsylvania, USA; NC State University, Raleigh, North Carolina, USA

**Keywords:** COVID-19, infection, influenza, saliva, virus, volatilome

## Abstract

The volatile metabolome, or volatilome, contains a wealth of information regarding individual health. Two parallel approaches can detect patterns of these volatile metabolites: chemometric analyses employing gas chromatography-mass spectrometry (GC-MS) or “noses” (trained animals or so-called electronic noses). While “noses” are exquisite odor discrimination tools, chemometric analyses are superior for determining the chemical identity of odorants related to disease conditions. Without identifying compounds altered by disease, it is difficult to uncover the responsible mechanism(s) and develop effective diagnostic tools. To understand how viral pathogens may alter the human volatilome, we collected saliva from healthy patients and patients with confirmed viral infections. Using headspace GC-MS analyses, we examined associations between 85 salivary volatiles and specific viral pathogens. Twenty-eight salivary volatiles were up- or downregulated (in relation to the healthy condition) by infection with one or more viral pathogens. Further examination indicated that salivary volatiles encode some level of viral specificity. In particular, SARS-CoV-2 infection produced a unique volatile pattern differing from several other common viral respiratory infections.

**IMPORTANCE:**

This research examined alterations of the salivary volatilome caused by confirmed respiratory virus infections. Results indicate that the salivary volatilome may specify the offending pathogen for improved clinical outcomes.

## INTRODUCTION

Both clinical and public health management of infectious diseases rely on the availability of accurate diagnostic tools. Rapid diagnostic evaluation is essential to facilitate appropriate management, including the provision of appropriate infection-specific therapies and isolation, and reduce onward transmission. On a larger scale, test/treat/track is a cornerstone of public health efforts to reduce the burden of infectious diseases on communities. Minor viral infections are typically self-limited, but often prompt potential bacterial infection concerns and therefore result in unnecessary antibiotic treatment. Finally, changing weather patterns and loss of animal habitats worldwide have raised heightened concerns for new zoonotic spillover events with pandemic potential. Pathogen-agnostic diagnostic tools that rapidly distinguish infected individuals with viral specificity would assist early detection and management of future pandemics. Simple to use, non-invasive diagnostic tools that do not require phlebotomy or other invasive sampling methods would be highly impactful.

There has been a longstanding interest in disease-associated volatile compounds as an infectious disease detection strategy. Ancient Greeks recognized the diagnostic value of abnormal bodily odors, and some older adults may recall a time when their family physician smelled patient urine as part of routine examinations. Odors of diphtheria (sweet, putrid), smallpox (sweet, pungent), tuberculosis (stale beer), and typhoid (baked bread) are long-recognized (1), as well as those of many metabolic disorders such as trimethylaminuria, diabetic ketosis, and maple syrup urine disease (2).

More recently, the volatilome has become a common diagnostic biomarker target (3). For example, tuberculosis is associated with distinct volatiles, which can be detected by trained scent-detection animals (4), and there are credible reports of trained dogs detecting human infections and malignancies (5–8). Using headspace gas chromatography-mass spectrometry, we have successfully demonstrated that alterations of body odor (expressed in feces or urine) result from infection (9), injury (10), inflammation (11), and immunization (12) in animals and from inflammation (13) and illness (14, 15) in humans. Recent reviews further illustrate the importance and interest in this research area (16–20).

Paired with sensor technology innovations, identifying disease-specific volatile patterns will produce easy-to-use, low-cost, reusable, and non-invasive diagnostics for use in low- and middle-income settings. Although recent disease-specific, proof-of-concept studies have strongly demonstrated the diagnostic value of the volatilome, few of these have addressed pathogen-specific identification. In contrast, many studies compare a single clinical disease against a healthy control group only. Our study evaluates whether viral infections with different pathogens may alter the human volatilome in distinct ways. In this work, we examined volatiles present in saliva from symptomatic subjects confirmed to have acute viral infections (including SARS-CoV-2, influenza A, adenovirus, rhinovirus, and respiratory syncytial virus), compared to healthy control subjects. In this study, we find that symptomatic viral infection, regardless of pathogen, has a characteristic volatile profile. Further, we provide evidence to support the ability to distinguish specific viral infections via non-invasive salivary volatiles testing.

## MATERIALS AND METHODS

### Human subject recruitment

Between May 2021 and February 2024, we collaborated with local clinical programs (Table 1) to enroll 303 febrile subjects with acute illness, ages 5–26 years. An additional 69 healthy volunteer subjects (ages 5–26 years) were recruited from the local community. The Institutional Review Board of Children’s Hospital of Philadelphia (21-018511) approved subject enrollment. Symptomatic patients were potentially eligible if they had documented current or recent (within 12 h of enrollment) fever, defined as body temperature ≥38.3°C. Exclusion criteria were history of immunocompromising conditions and body weight less than the third percentile for age and sex. Our study was explained to eligible patients and families to obtain informed consent (subjects 18–26 years, and parents of 5–17 years subjects) and assent (subjects 7–17 years) prior to enrollment.

**TABLE 1:** Characteristics of study population

Characteristic	Afebrile and healthy (*N* = 69)	Febrile (*N* = 279)	Overall (*N* = 348)
Age			
Mean (SD)	15.9 (7.1)	9.2 (4.2)	10.5 (5.6)
Median (min, max)	16.0 (5.0, 26.0)	8.0 (5.0, 22.0)	8.0 (5.0, 26.0)
Sex			
Female	38 (55.1%)	162 (58.1%)	200 (57.5%)
Male	31 (44.9%)	117 (41.9%)	148 (42.5%)
Race			
American Indian/Alaska Native	1 (1.4%)	0 (0%)	1 (0.3%)
Asian	7 (10.1%)	13 (4.7%)	20 (5.7%)
Black or African American	13 (18.8%)	115 (41.2%)	128 (36.8%)
More than one race	4 (5.8%)	7 (2.5%)	11 (3.2%)
Other	4 (5.8%)	34 (12.2%)	38 (10.9%)
White	40 (58.0%)	101 (36.2%)	141 (40.5%)
Unknown or not reported	0 (0%)	9 (3.2%)	9 (2.6%)
Ethnicity			
Hispanic or Latino	8 (11.6%)	45 (16.1%)	53 (15.2%)
Not Hispanic or Latino	60 (87.0%)	191 (68.5%)	251 (72.1%)
Unknown or not reported	1 (1.4%)	43 (15.4%)	44 (12.6%)
Enrollment site			
CHOP Center for Human Phenomic Science^*a*^	69 (100%)	0 (0%)	69 (19.8%)
CHOP emergency and urgent care sites	0 (0%)	262 (93.9%)	262 (75.3%)
CHOP outpatient offices	0 (0%)	4 (1.4%)	4 (1.1%)
Student health clinics	0 (0%)	13 (4.7%)	13 (3.7%)

^*a*^ CHOP, Children's Hospital of Philadelphia.

Febrile subjects were given COVID-19 tests as well as a viral respiratory panel for influenza A, influenza B, RSV, adenovirus, rhinovirus, human metapneumovirus, parainfluenza 1, parainfluenza 2, parainfluenza 3, coronavirus OC43, coronavirus NL63, coronavirus HKU1, and coronavirus 229E. The majority of febrile patients also received a rapid strep test. Afebrile (presumed healthy) subjects also received initial COVID-19 tests. Negative results prompted SARS-CoV-2 antibody tests to assess prior exposure. Supplemental Methods detail these diagnostic tests.

### Sample collection

Following a sip of water, we collected saliva using a Salivette collection kit (Sarstedt, Newton, NC, USA). This method uses a highly absorbent cylindrical-shaped pad to provide a saliva specimen in approximately 2 min. Participants held the pad between their lower jaw and cheek for 2 min without biting down. Centrifugation of Salivette pads eluted saliva into a labeled 10 mL sterile storage tube for frozen preservation at −80°C until transfer to Monell Chemical Senses Center for chemical analyses. Not all enrolled subjects produced sufficient saliva volumes needed for analysis (Table 2). In total, 309 of the 372 subjects produced saliva samples (representing 264 febrile and 45 healthy subjects).

**TABLE 2:** Number of samples and age/sex demographics for saliva samples collected from subjects with the following confirmed pathogens or diagnosed syndromes: COVID-19, influenza A, rhinovirus, adenovirus, respiratory syncytial virus (RSV), viral gastroenteritis, viral pharyngitis, viral syndrome, other, and healthy^*a*^

Characteristic	Healthy (*N* = 45)	Covid (*N* = 12)	Influenza (*N* = 44)	Rhino (*N* = 13)	Adeno (*N* = 10)	RSV (*N* = 7)	Gastro (*N* = 6)	Pharyn (*N* = 10)	Syndrome (*N* = 33)	Other (*N* = 57)
Age										
Mean (SD)	14.5 (7.3)	11.2 (4.2)	9.6 (3.6)	11.0 (5.2)	7.8 (2.2)	5.6 (0.5)	10.0 (2.5)	7.1 (0.9)	9.4 (3.7)	10.4 (4.3)
Median (min, max)	13.0 (5, 26)	12.0 (5, 17)	9.0 (5, 18)	9.0 (5, 21)	7.0 (6, 13)	6.0 (5, 6)	9.5 (7, 13)	7.0 (6, 8)	8.0 (5, 18)	9.0 (5, 20)
Sex										
Female	22 (48.9%)	7 (58.3%)	24 (54.5%)	6 (46.2%)	3 (30.0%)	0 (0.0%)	4 (66.7%)	8 (80.0%)	23 (69.7%)	37 (64.9%)
Male	23 (51.1%)	5 (41.7%)	20 (45.5%)	7 (53.8%)	7 (70.0%)	7 (100%)	2 (33.3%)	2 (20.0%)	10 (30.3%)	20 (35.1%)

^*a*^ Among these, four samples were determined to be outliers by principal components analysis: one healthy, one viral pharyngitis, and two other.

### Chemical and statistical analyses of saliva

Supplemental Methods detail headspace analyses, chromatographic processing, and statistical analyses. Briefly, saliva samples (250 mg target mass, accurately determined) were fortified with an internal standard solution of acetophenone-d5. Headspace volatiles captured by solid-phase microextraction (SPME) were analyzed by gas chromatography-mass spectrometry (GC-MS) during the period of 12 January 2022 to 1 March 2024. Peak responses were normalized to the internal standard, yielding a normalized (semi-quantitative) concentration in parts-per-billion. Tentative compound identifications were made by comparison to the NIST Standard Reference Database (21) and confirmed by comparison to known standards when available.

We first used partial least squares discriminant analysis (PLS-DA) to discriminate between healthy and febrile conditions using independent variables (salivary volatiles, subject sex, and age) from subjects with confirmed viral diagnoses and healthy subjects with no evidence of prior SARS-CoV-2 infection. Variable importance (VIP) scores were examined to determine which predictors contributed most to diagnostic discrimination (22). The analysis was repeated a second time using only those predictors with VIP scores greater than 0.8. Next, we used this PLS-DA model to predict the condition (healthy or febrile) of subjects diagnosed with viral gastroenteritis, viral pharyngitis, respiratory viral syndrome, or “other” viral condition and healthy subjects with a positive SARS-CoV-2 antibody test (or no valid immunoassay test).

We then examined how each PLS-DA predictor varied between healthy and febrile conditions using repeated measures analysis of variance with linear contrasts. Finally, we conducted a hierarchical cluster analysis to examine relationships among the five molecularly confirmed viral infections (COVID-19, influenza A, adenovirus, rhinovirus, and RSV) as determined by mean (by diagnosis) values of 51 salivary volatiles identified in the final PLS-DA model.

## RESULTS

One of the 69 subjects in the healthy subject recruitment group tested SARS-CoV-2 positive by PCR and 12 others tested positive for SARS-CoV-2 antibodies by immunoassay (indicating prior exposure to the virus). Two subjects did not have valid immunoassay results. Among 67 healthy subjects with a valid immunoassay test, 54 healthy subjects had no indication of exposure to SARS-CoV-2 (80.6%).

In total, 309 of the 372 subjects produced saliva samples (representing 264 febrile and 45 healthy subjects). Diagnostic tests eliminated 72 of the 264 febrile subjects diagnosed with a single or multiple bacterial infection. Remaining saliva samples represented either a viral (*N* = 192) or healthy (*N* = 45) condition. Among samples from subjects with viral infection, 86 represented one of the five confirmed infections: SARS-CoV-2, influenza A, adenovirus, rhinovirus, or respiratory syncytial virus (RSV). Unconfirmed viruses (106 samples) were assigned as: viral gastroenteritis, viral pharyngitis, respiratory viral syndrome, or “other” viral condition (Table 2). Examples of “other” diagnoses were Epstein-Barr virus, metapneumovirus, parainfluenza, multiple viral infections, combined viral and bacterial infections, unspecified upper respiratory illness, and so forth.

Feature filtering of chromatographic data yielded 85 normalized salivary volatile features available for statistical analyses. Following removal of four samples (one healthy, one viral pharyngitis, and two “other”) observed as outliers by principal components analysis, the final data represented: 86 confirmed viral diagnoses, 103 unconfirmed, and 44 healthy (34 healthy with no evidence of prior SARS-CoV-2 infection).

**Fig 1:**
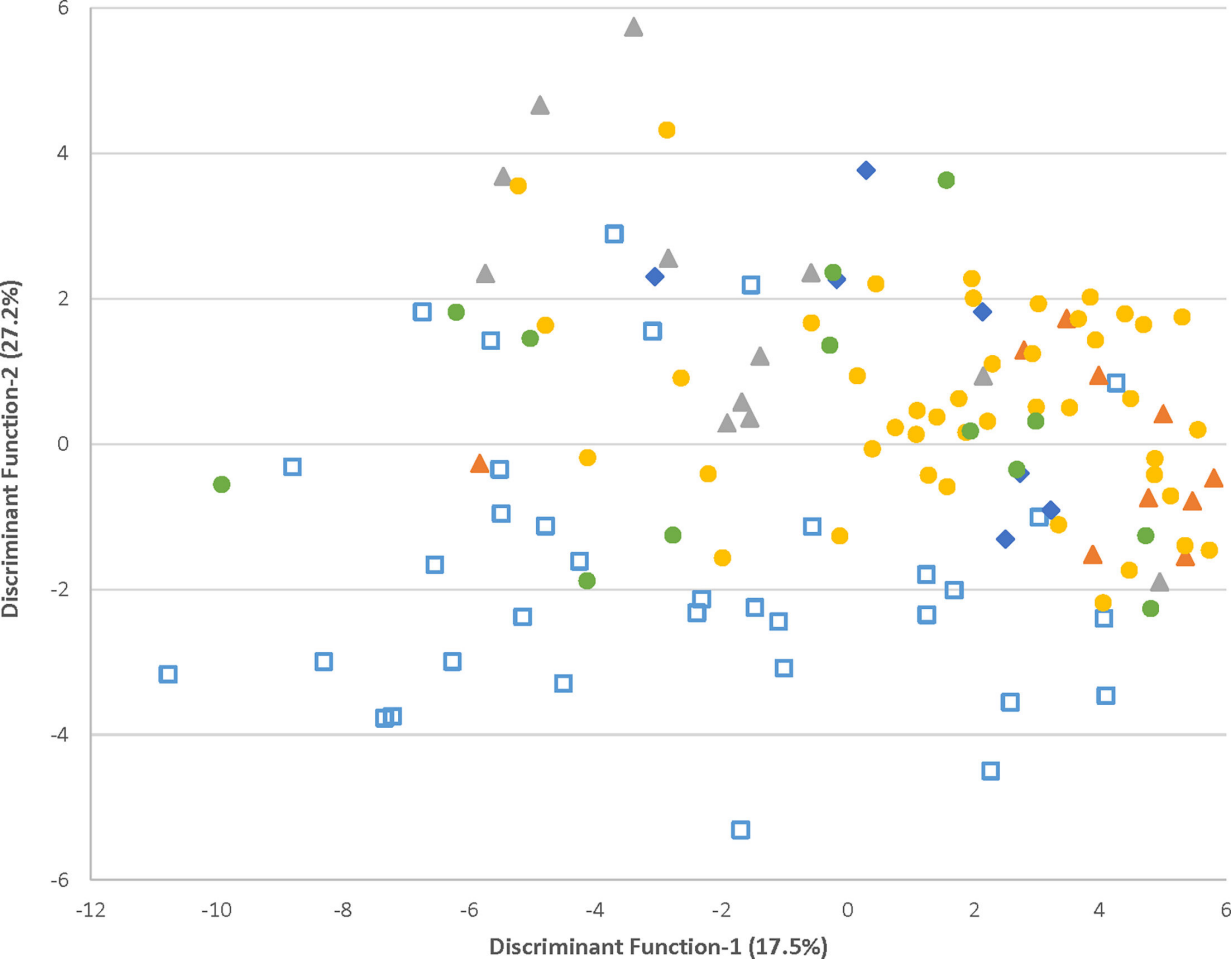
Canonical score plot of confirmed viral infections and healthy using two factors determined by the final PLS-DA model depicting 94% correct discrimination of viral infections (solid markers) and 62% of healthy (open squares). Orange triangles, adenovirus; gray triangles, COVID-19; orange circles, influenza A; diamonds, respiratory syncytial virus (RSV); and green circles, rhinovirus.

**Fig 2:**
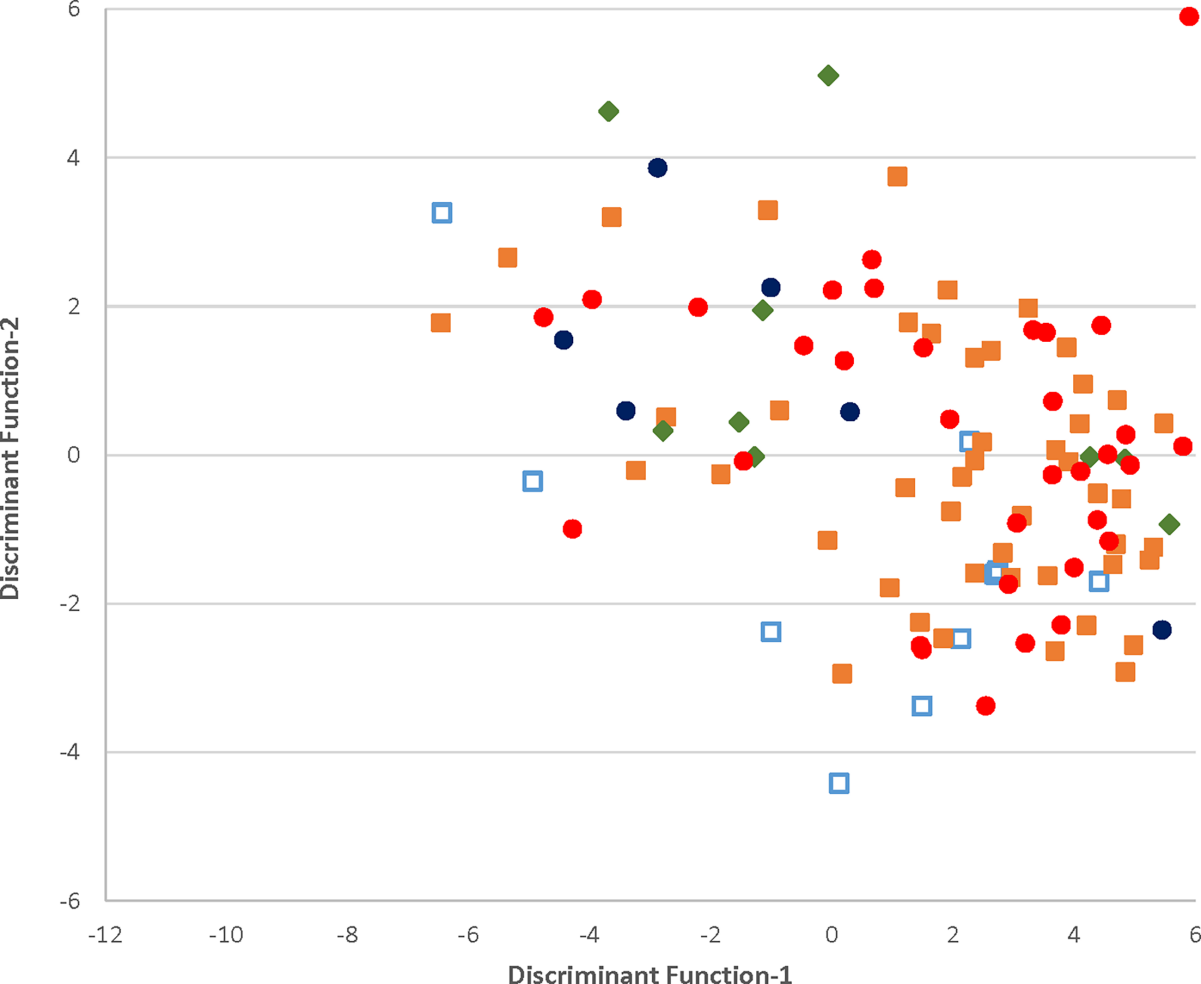
Canonical score plot (same scale as Fig. 1) of PLS-DA predictions for unconfirmed viral infections and healthy subjects with prior or unknown exposure to SARS-CoV-2 depicting 94% correct classification of viral infections (solid markers) and 60% of healthy (open squares) from samples excluded from model building. Orange squares, other; black circles, viral gastroenteritis; diamonds, viral pharyngitis; and red circles, viral syndrome.

The final PLS-DA model (using 86 subjects with a confirmed viral diagnosis and 34 healthy subjects with no evidence of prior SARS-CoV-2 infection) incorporated 51 salivary volatiles and subject age (at time of sample collection) to discriminate between healthy and febrile conditions (Fig. 1). The model successfully classified 81 of the 86 (94%) subjects with viral infections and 21 of the 34 (62%) healthy subjects. Our PLS-DA model also attributed 97 of the 103 (94%) samples from subjects with viral syndromes, and 6 of the 10 (60%) samples collected from healthy patients having prior exposure to SARS-CoV-2 (Fig. 2).

Having demonstrated that patterns of volatiles were altered generally by viral infection, we performed repeated measures analysis to examine how individual volatiles from the PLS-DA model differed among specific diagnoses (healthy, COVID-19, influenza A, adenovirus, rhinovirus, and RSV). Importantly, a highly significant diagnosis*volatile effect (*P* < 0.0001) strongly supported our central hypothesis that individual volatiles differ according to diagnosis. This analysis also revealed significant diagnosis (*P* = 0.0002) and volatile (*P* < 0.0001) effects. Among 51 volatile metabolites employed in the final PLS-DA model, univariate ANOVA tests revealed that 34 varied significantly among at least one health condition (Table 3).

**TABLE 3:** Thirty-four salivary volatile metabolites found to differ among at least one health condition (healthy and/or confirmed viral infection)^*a*^

Time	Volatile metabolite	Source	VIP	PROB	α	Healthy
5.45	**2-Pentanone**	D, M	1.182	0.00016	0.00392	X
6.30	5-Ethyl-2,2,2-trimethyl heptane	D	0.764	0.0075	0.02647	X
6.50	2,2,7,7-Tetramethyl octane	D	1.487	0.0018	0.01569	X
6.72	**Ethyl butyrate**	D, H, M	0.814	0.0028	0.01863	X
7.15	**3-Hexanone**	D, M	0.821	0.0091	0.02745	X
7.37	3,7-Dimethyl decane	D	0.842	0.0028	0.01765	X
7.51	3-Methyl-5-propyl nonane	D	0.968	0.0029	0.02059	X
7.89	**Hexanal**	D, H, N	1.097	0.00004	0.00098	X
8.47	**2-Methyl-2-pentanol**	M	1.023	0.00083	0.01176	X
9.05	Unknown (*m/z* = 103)	Unknown	0.753	0.00027	0.00686	X
9.66	**1-Butanol**	D, M	0.731	0.00022	0.00588	X
9.84	**3-Heptanone**	D, M	0.728	0.00044	0.00980	X
10.31	**2,6-Dimethyl-4-heptanone**	D	0.653	0.0098	0.02843	
10.68	**2-Heptanone**	D, H, M	0.760	0.0041	0.02353	
10.82	**Pyridine**	D, M	0.898	0.0012	0.01471	X
11.01	**2-Methyl-2-hexanol**	M	1.093	0.00087	0.01275	X
11.35	4-Methyl-2-heptanone	M	0.749	0.0029	0.01961	
11.55	**3-Methyl-1-butanol**	M	0.829	0.0333	0.03333	X
11.70	**2-Hexanol**	D, M	1.001	0.0035	0.02255	X
12.38	2,5-Dimethyl-2-hexanol	D, H	1.089	0.00027	0.00784	X
12.57	**1-Pentanol**	D, M	1.033	0.00013	0.00294	X
13.60	3-Methyl-3-heptanol	Likely D	1.166	0.00030	0.00882	X
13.67	**Octanal**	D, H, M	0.881	0.0049	0.02451	X
13.89	**Cyclohexanone**	D, M	0.696	0.00056	0.01078	
14.09	**3-Hepten-2-one**	D, H	1.334	0.00008	0.00196	X
14.40	2-Methyl-2-octanol	Likely D	1.049	0.00017	0.00490	X
15.43	**1-Hexanol**	D, H, M	0.625	0.0070	0.02549	X
15.77	**2-Cyclopenten-1-one**	M	0.757	0.0112	0.03039	X
20.18	**Benzaldehyde**	D, H, M	1.501	0.0012	0.01373	X
25.53	**2-Ethyl butyric acid**	D, H, M	0.834	0.0191	0.03235	
32.15	N-Acryloylmorpholine	Plasticizer	0.944	0.0108	0.02941	X
33.65	2,4-Dimethylquinoline	Environ	0.689	0.0144	0.03137	X
34.64	**Caprolactam**	D, M	0.993	0.0033	0.02157	X
40.58	**Dibutyl phthalate**	D, M	0.749	0.0018	0.01667	

^*a*^ Time is the chromatographic retention time. Source describes known origins of stated compounds; with “D” indicating a known dietary source (23), “H” indicating a known human metabolism role (23), and “M” indicating a known microbial source (24). VIP is the variable importance projection score from the final PLS-DA model (22). PROB describes the ANOVA probability that salivary concentration of at least one condition varies significantly from the others (including the healthy condition). Alpha describes the test criterion determined by controlling for false discovery rate (25). Those 28 metabolites indicated with an “X” under the Healthy column indicate up- or downregulation from the healthy condition for at least one viral infection determined by linear contrast. Tentative compound identifications were made by comparison of mass spectra to the NIST Standard Reference Library (21). Compound identifications confirmed by comparison to known standards are given in bold.

**Fig 3:**
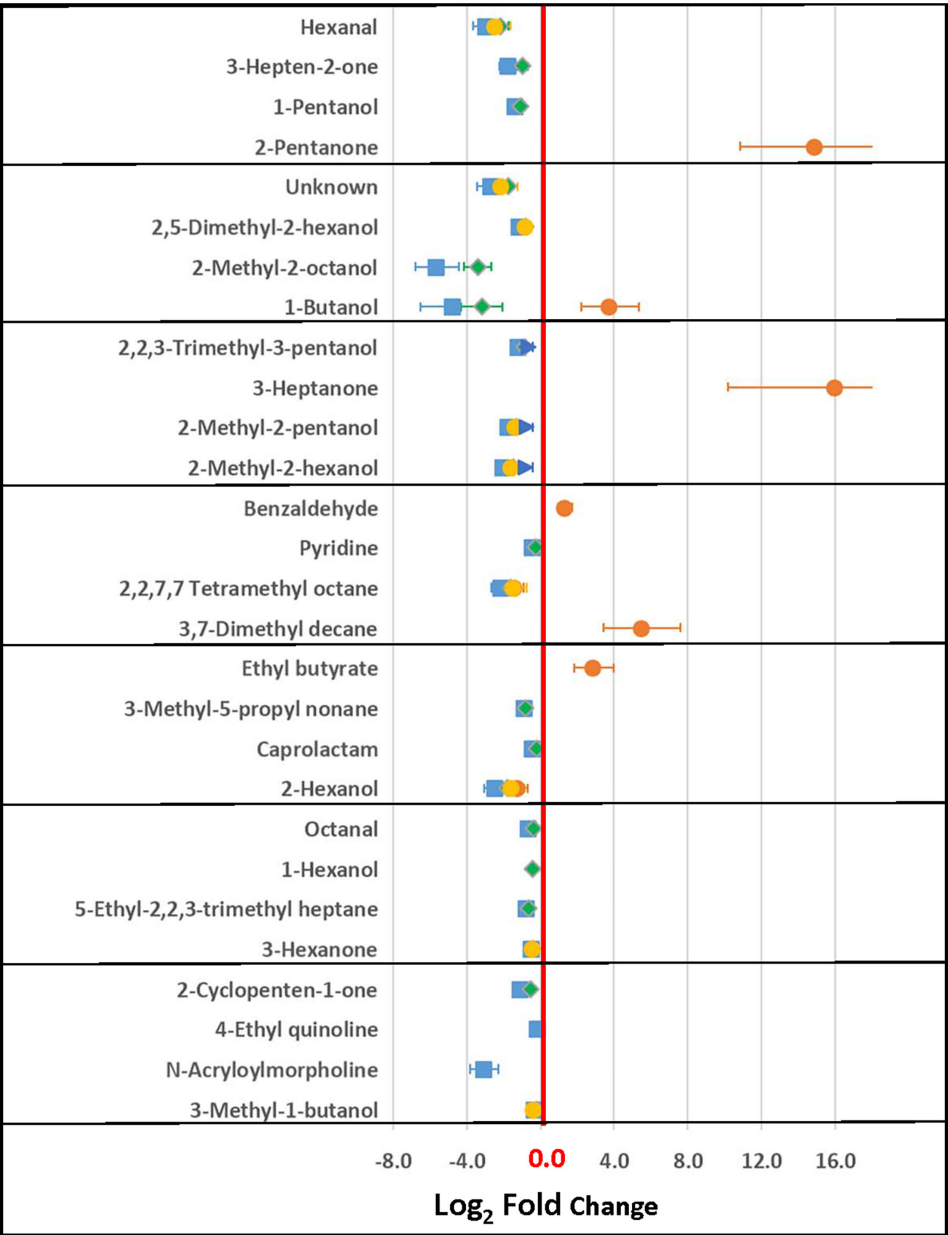
Log_2_ fold change for 28 salivary volatiles differing from the healthy condition (red line). Only significant differences from healthy are presented. Horizontal lines represent standard error of the mean. Triangles, rhinovirus; yellow ovals, respiratory syncytial virus (RSV); diamonds, influenza A; orange ovals, COVID-19; and squares, adenovirus.

Using linear contrasts, we next examined these 34 salivary volatiles for differences between each viral and healthy condition. Salivary concentrations of 28 of these volatiles were either significantly increased or decreased as compared to healthy subjects (Fig. 3). In most cases, concentrations of salivary volatiles decreased in infected subjects (as compared to healthy subjects). Only six instances of upregulation were observed, and each was associated with a COVID-19 diagnosis (Fig. 3). We found that no single metabolite was altered (relative to healthy) in all five diagnosed viral infections. However, concentrations of both 2-methyl-2-hexanol and 2-methyl-2-pentanol significantly decreased in all conditions except COVID-19 (Fig. 3). Concentrations of 2-hexanol and a branched alkane also decreased in all conditions except rhinovirus. Twenty-two of these 28 salivary volatiles were downregulated (as compared to healthy) in subjects diagnosed with adenovirus infection and 21 by influenza virus infection. RSV infection reduced 9 of the 28 volatile metabolites. Perhaps unsurprisingly (due to its mild symptomology), rhinovirus infection was associated with only modest changes in salivary volatiles, with just three notable differences compared to healthy controls, including 2-methyl-2-hexanol and 2-methyl-2-pentanol.

**Fig 4:**
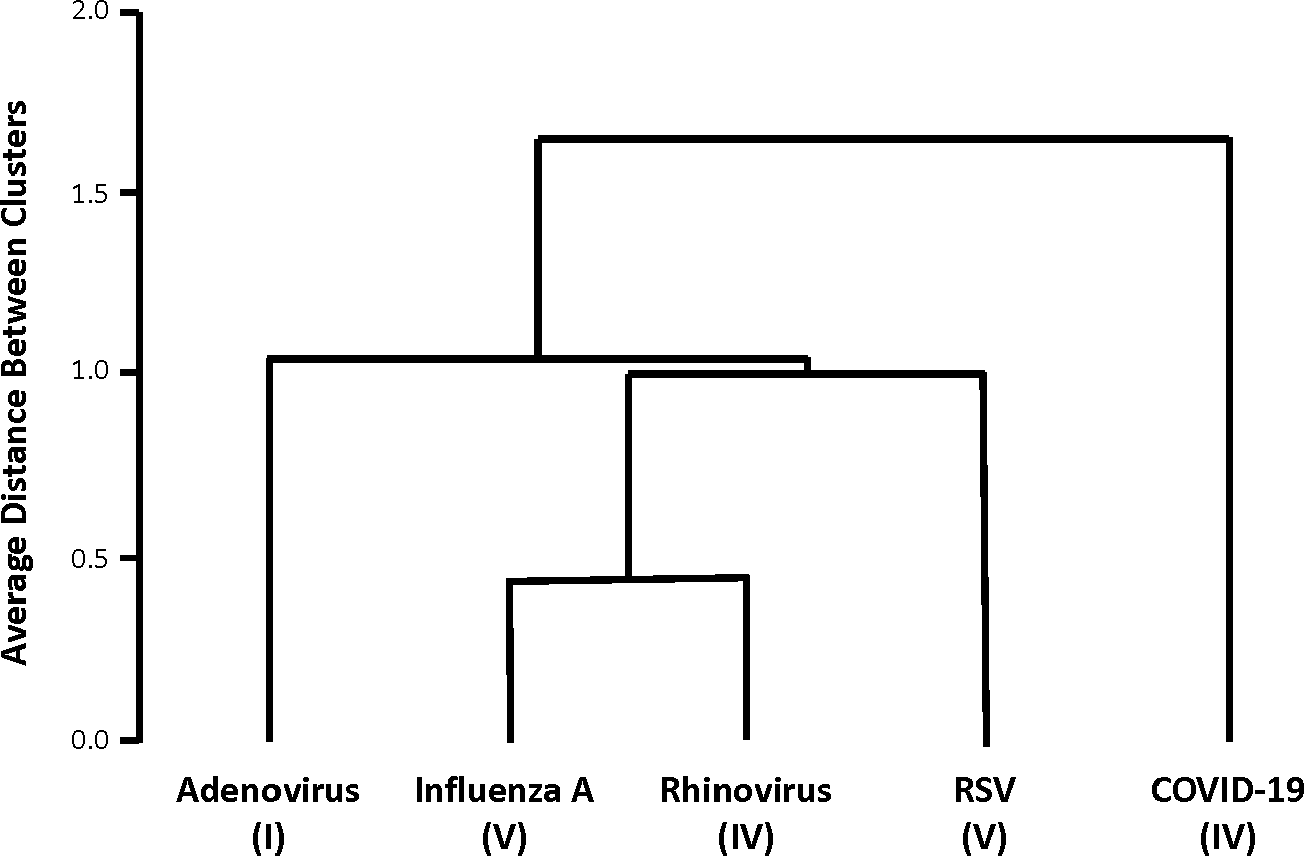
Dendrogram resulting from hierarchical cluster analysis of mean salivary volatile responses from five confirmed viral infections. Cluster analysis revealed the critical distance to be approximately 0.5. Baltimore virus classification is given for each virus: (I) double-stranded DNA; (IV) positive-sense, single-stranded RNA; and (V) negative-sense, single-stranded RNA (26).

Finally, hierarchical cluster analysis of five confirmed viral infections demonstrated that only influenza A and rhinovirus infections had similar volatile patterns (i.e., cluster analysis assigned them to the same cluster; Fig. 4). Aside from this combined cluster, patterns of salivary volatiles differed between other clusters (viruses). The volatile pattern associated with COVID-19 was most unique (as evidenced by its average distance to other clusters; Fig. 4).

## DISCUSSION

In our study, we find that viral infection in humans has a distinct salivary volatile profile regardless of pathogen. Although no individual salivary volatile was sufficiently diagnostic, our PLS-DA model performed well to broadly diagnose viral infection with extremely high sensitivity (94%). Encoded in these same volatile patterns, we find strong evidence that several viral pathogens produce distinct salivary volatile profiles (Fig. 4). Most notably, acute COVID-19 was associated with a markedly different volatile profile as compared to other confirmed viral infections. Specifically, a COVID-19 diagnosis was uniquely associated with increased levels of discriminatory volatiles, as compared to other respiratory viral pathogens that all featured decreased salivary volatiles (Fig. 3). This distinctive alteration of volatile metabolites associated with infection with the SARS-CoV-2 virus may likely contribute to documented abilities of dogs to identify COVID-19 with high levels of sensitivity and specificity (27, 28). In contrast, we find that rhinovirus infection is associated with relatively modest changes, and its pattern of salivary volatiles is indistinguishable from influenza infection (Fig. 4).

The biological origin of viral-associated salivary volatile changes is unclear. No obvious association with virus structure and replication strategy (as described by Baltimore virus classification [26]) is evident (Fig. 4). Volatile patterns observed across pathogens in our study suggest that the immune response to infection may help, at least in part, shape these volatile profiles and uniqueness of COVID-19. For example, SARS-CoV-2 is known to be highly immunogenic and acute infection is associated with increased levels of pro-inflammatory cytokines, such as IL-1β and IL-6 (29). Differences in cytokine expression are observed during both early and late phases of illness produced by infection with RNA viruses (30). Recently, specific and reproducible alterations of urinary volatiles associated with pathogen-specific activation of immune pathways were demonstrated in an animal model (31).

Differences in immunological memory of COVID-19 may have also influenced the unique volatile response associated with COVID-19. Whereas many subjects in this study were unlikely to have been previously exposed to SARS-CoV-2 (our own data indicate that 80% of the healthy cohort had no evidence of prior COVID-19 exposure at sample collection), they most certainly would have had many encounters with other viruses. A recent study demonstrated that by 2 years of age, 100% of children have experienced at least one exposure to rhinovirus and the exposure rates by age 2 were 66% for RSV, 59% for adenovirus, and 27% for influenza virus (32). Thus, it is plausible that unique alteration of the salivary volatilome associated with COVID-19 represents a difference in viral infection experience.

Importantly, viruses have evolved to target the host’s metabolic pathways (29). For example, different viruses alter glutaminolysis (process of using glutamine to produce tricarboxylic acid cycle intermediates) and lipid metabolism (storage or breakdown of lipids) at different points of these pathways (33). Many of the salivary volatiles downregulated by viral infection in this study are aldehydes, ketones, and alcohols known to be involved with some aspect of human metabolism (Table 3). Aldehydes are known to be involved in glycolysis and lipid metabolism, many ketones are involved in ketogenesis, and alcohols can result from the metabolism of hydrocarbons (34).

The oral microbiome also shapes, at least in part, the salivary volatilome. The oral cavity is home to over 700 species of bacteria, as well as fungi, viruses, and protozoa (35). Among the principal microbes are bacteria: *Streptococcus*, *Lactobacillus*, and *Campylobacter*; fungi: *Candida*, *Fusarium*, and *Aspergillus*; and protozoa: *Entamoeba gingivalis* and *Trichomonas tenax*. These same microbes have been previously linked with nearly all salivary volatiles associated with viral infection in this study (24), suggesting a plausible link between the supporting microbiome and volatilome alterations caused by viral infection. For example, a decrease in species richness (α-diversity) has been observed in saliva and stool as a result of infection with SARS-CoV-2 (36), whereas the oral microbiome is thought to be stable following influenza infections (37). In fact, species richness of the oral microbiome is predictive of COVID-19 status (38). Since most viral infection-associated volatiles appear to be reduced (rather than increased), perhaps alterations in diet (due to illness-associated anorexia or pharyngitis) or mouth breathing (in the setting of nasal congestion from upper respiratory infection) negatively influence oral microbiome composition or metabolic activity.

Despite the strengths of this study, which include a relatively novel approach of comparing volatilomes of several different viral pathogens, our study has a number of limitations. Among these was the age difference between healthy subjects and those with viral infections (Table 1). Because healthy subject age skewed high as compared to viral subjects, we sought to evaluate this effect by employing age as a covariate in statistical analyses. The lack of a significant volatile*age effect in repeated measures ANOVA indicates that disparity of ages among diagnoses had little impact on the distribution of volatiles in saliva. Subject sizes among viral classification groups were an additional limitation, such that several confirmed viral groups were relatively underpowered.

Regardless of these limitations, our results provide ample evidence that it is possible to discriminate certain viral pathogens using salivary volatiles. In contrast to blood, urine, and other volatile substrates such as breath, saliva is extremely simple and safe to collect, even in a home environment. Accordingly, saliva is emerging as an important diagnostic fluid for molecular testing to evaluate viral shedding (39). Our study provides additional support for saliva as a rich source of diagnostic volatile metabolites. Although the performance characteristics of our study suggest relatively poor specificity in ruling out viral infection (4/10 = 40% false positives in a small test set of healthy subjects), the high sensitivity of salivary volatiles suggests promise for the ongoing development of a pathogen-agnostic salivary test.

Limited availability of SARS-CoV-2 specific PCR primers hampered the early response to COVID-19 in the United States, leading to substantial under-testing. At the height of the pandemic response, massive testing campaigns (e.g., every hospital admission) frequently strained personnel and resources. A simple screening test, even with insufficient specificity, could help reduce this testing burden. Early diagnoses will also have strong utility for limiting disease transmission and streamlining access to therapeutics. In particular, the burgeoning field of antiviral drug development will demand easily accessible, rapid, and specific testing. Our study indicates that the salivary volatilome not only encodes broad information regarding presence of viral infection, but may also improve clinical outcomes by specifying offending pathogens.

## Data Availability

Data underlying this paper are available on request.

## SUPPLEMENTAL MATERIAL

Supplemental Material msphere.00222-26-s0001.docx Supplemental methods and table.

ASM does not own the copyrights to Supplemental Material that may be linked to, or accessed through, an article. The authors have granted ASM a non-exclusive, world-wide license to publish the Supplemental Material files. Please contact the corresponding author directly for reuse.
