# Supplementary material for: Viral infections alter the human salivary volatilome
Source: mSphere. 2026 May 11;11(6):e00222-26. doi: 10.1128/msphere.00222-26 (PMC13317186; doi:10.1128/msphere.00222-26)
Supplement: Supplemental Material — Supplemental methods and table. [file msphere.00222-26-s0001.docx]

**SUPPLEMENTAL METHODS**

**Diagnostic Testing.** Two respiratory mucosal samples (anterior nasal) were collected by inserting a nylon-flocked nasal swab one-half inch into naris and circling 5 times on each side. One sample was immediately analyzed by PCR for presence of SARS-CoV-2 infection. The second of the respiratory mucosal swabs was stored at -80 ^o^C. For the majority of participants, a Rapid Strep Test (OSOM Strep A Test, Sekisui Diagnostics, Burlington, MA) was conducted to identify febrile conditions caused by bacterial infection. Blood samples were collected from healthy participants for SARS-CoV-2 antibody testing using CHEMIFLEX methodology on the Architect i2000 immunoassay analyzer (Abbott; Chicago, IL, USA) to test for prior SARS-CoV-2 infection. For healthy participants, the second swab remained stored at -80 ^o^C and were not further used.

For febrile participants with negative tests for SARS-CoV-2, Strep, or any additional clinical testing, the second nasal swabs were submitted for viral respiratory panels using magnetic bead technology for nucleic acid extraction (Roche MagNA Pure LC 2.0 or Qiagen EZ XL Advanced) and PCR (Applied Biosystems Quantstudio Dx). Viruses included influenza A, influenza B, RSV, adenovirus, rhinovirus, human metapneumovirus, parainfluenza 1, parainfluenza 2, parainfluenza 3, coronavirus OC43, coronavirus NL63, coronavirus HKU1, and coronavirus 229E. Febrile participants without definitive viral diagnoses were given clinical diagnoses defined by symptomology: viral gastroenteritis (nausea, vomiting, diarrhea, abdominal pain), viral pharyngitis (pharyngeal pain and swelling), respiratory viral syndrome (cough, sputum production), or other.

**Headspace Analyses of Saliva Samples**. Samples were periodically transferred to the chemical analysis laboratory at Monell where they were maintained at -80 ^o^C until thawed at room temperature for analysis. Data from the 30 unique chromatographic runs were grouped into “batches” (post hoc) to represent unique, short-term periods of analyses (Table S1). The elapsed number of days constituting a batch ranged from two to fifteen (mean = 5 days) for 11 batches identified over 779 days of the study. Pauses between batches ranged from 19 to 158 days (mean = 74 days). Batch information was used for feature filtering (see below).

Saliva was transferred with a 250 μL volumetric pipette to tared, 20-mL glass headspace vials and the mass determined. Volumetric transfer typically yielded the target mass of 250 mg, but actual sample masses ranged from approximately 100 to 300 mg due to sample availability and variability of saliva viscosity. Samples were proportionally fortified with a 2.48 μg/mL solution of acetophenone-d5 such that the final internal standard concentration in saliva was a constant 248 ng/g for sample regardless of sample mass. Capped vials (septa crimp-caps) were subjected to gas chromatography-mass spectrometry analyses employing solid-phase microextraction (SMPE) headspace collection. Quality control samples consisted of empty vials and vials fortified with 10.0 μL of the 6.17 μg/mL internal standard solution. Automated headspace collection was achieved with a Thermo Scientific Tri-Plus RSH autosampler (Waltham, MA, USA) equipped with a 1.10 mm DVB/Carbon-WR/PDMS Arrow® SPME tool (120 μm thickness; 20 mm phase length; Restek Corporation, Bellefonte, PA, USA). For each analysis, samples were incubated at 37 ^o^C for 10 min in the RSH incubation station while the fiber was pre-conditioned for 10 min at 260 ^o^C in the RSH conditioning station. Volatile collection was made at 37 ^o^C for 10 min while spinning at 1000 rpm in the RSH sampling station. Collected volatiles were thermally desorbed from the Arrow® at 230 ^o^C in the GC injection port for 2.0 min, followed by thermal post-injection fiber cleaning at 260 ^o^C for 15 min in the RSH conditioning station.

Chromatographic analyses were performed using a Thermo Scientific ISQ single quadrapole GC/MS (Waltham, MA, USA) equipped with a 30 m x 0.25 mm Stabilwax-DA capillary column (Restek Corporation, Bellefonte, PA, USA) using helium carrier gas flowing at 1.1 mL/min in constant flow mode (with vacuum compensation). Splitless injections were made with a 1.0 min split time (split flow of 25.0 mL/min). The initial oven temperature was 40 ^o^C (held for 2.0 min) and increased at a rate of 5.0 ^o^C/min until the final temperature of 230 ^o^C (held for 2.0 min). Detection was achieved with a Thermo Scientific ISQ single quadrapole mass spectrometer (Waltham, MA, USA), operating in scan mode from 33 to 400 m/z after a 5.0 min solvent delay. The chromatographic runtime was 42 min and the scan rate was 3 Hz.

All chromatographic data were exported to NetCDF format for baseline correction and peak alignment processing using Metalign software^TM^ (Lommen, 2009). The resulting multivariate data (consisting of all mass spectrometric responses exceeding a defined threshold at each scan event) were then processed using the MSClust tool (Tikunov et al., 2012). MSClust permits unsupervised determination of chromatographic peaks and yields a single response (corresponding to peak abundance) for each volatile metabolite. The process initially revealed 213 unique peaks from saliva samples.

Feature filtering was conducted to eliminate peaks that could not be attributed to saliva or were not present in levels exceeding baseline noise. This was achieved by examining peak responses in a batch-wise fashion (Table S1). First, mean peak responses and standard deviations were calculated for all peak responses in QC samples containing internal standard only. From these, the 95^th^ percentile of the t-distribution was then calculated for each peak. Next, median values for each peak response from saliva were compared to the 95^th^ percentile value determined from the QC samples. Features were retained when the median peak response from saliva exceeded the 95^th^ percentile value of the QC samples in at least two of the 11 batches (the internal standard peak response was excluded from this comparison). This process reduced the number of saliva features to 103. The number of retained features was further refined through manual examination to eliminate silica-based compounds arising from the vial septa. Ultimately, 85 saliva peaks (in addition to the internal standard) were retained.

**Statistical Analyses**. The multivariate data were first subjected to principal components analysis (PCA) to visually identify outlier samples exhibiting undue influence or leverage in residual plots using Unscrambler® (CAMO Software; Oslo, Norway). Four samples (one healthy, one viral pharyngitis and two “other”) were determined to be outliers and removed from the data set. For the purpose of modelling viral status (yes or no), subjects with a confirmed viral diagnosis and healthy subjects with no evidence of prior SARS-CoV-2 infection were assigned to the model group. The model group consisted of 86 febrile and 34 healthy subjects. Unconfirmed febrile subjects diagnosed with viral gastroenteritis, viral pharyngitis, respiratory viral syndrome, or “other” viral condition and healthy subjects with a positive SARS-CoV-2 antibody test (or no valid PCR test) were assigned to the prediction group. The prediction group consisted of 103 febrile and 10 healthy subjects

Partial Least Squares Discriminant Analyses (PLS-DA) were conducted in SAS using the PLS procedure with one-at-a-time cross-validation. The predictors used for PLS-DA model building to discriminate viral and healthy patients included the normalized peak responses of 85 volatiles, sex as assigned at birth (coded 1 or 0), and subject age at the time of sample collection. PLS-DA was performed on the model set using the viral response (0 = healthy and 1 = viral condition) and all predictors. The number of discriminant factors yielding the minimum predicted residual sum of squares were determined with leave-one-out cross validation. Details regarding model effect loadings and variable importance (VIP scores) were examined to determine which predictors contributed most to diagnostic discrimination (Wold, 1994). The analysis was repeated a second time using only those predictors with VIP scores greater than 0.8. This final model was used to predict the viral responses of samples from the prediction set.

Repeated measures analysis of variance (ANOVA) was used to evaluate how individual predictors from the final PLS-DA model varied according to confirmed diagnosis (COVID-19, influenza A, adenovirus, rhinovirus, respiratory syncytial virus (RSV), or healthy) using the generalized linear model (PROC GLM) in SAS. Normalized peak responses were considered repeated measures of the variable “volatile”. Diagnosis, age, and age*diagnosis were between-subjects effects; while volatile, volatile*diagnosis, volatile*age, and volatile*age*diagnosis were the within-subjects effects. When volatile*age and volatile*age*diagnosis effects were not significant (indicating that patterns of volatiles did not differ according to age), the analysis was repeated without age in the model. Upon discovery of a significant diagnosis*volatile effect, indicating that at least one volatile was associated with one or more diagnosis, univariate analyses were employed to determine which volatiles differed according to diagnosis.

For univariate analyses (separate ANOVA for each volatile metabolite), the false discovery rate (FDR) controlling procedure was employed to account for the many univariate ANOVA tests (Benjamini and Hochberg, 1995). Post hoc comparisons among the diagnoses were performed using the pdiff option in SAS. For those individual volatiles demonstrating a probability less than FDR-adjusted decision criterion (α), the specific relationship between each pathogen (COVID-19, influenza A, adenovirus, rhinovirus, and RSV) and the healthy condition were determined by linear contrast using the ESTIMATE statement in the GLM procedure. Fold changes (Log2) were calculated for each instance where the normalized peak response differed significantly between a confirmed viral diagnosis and the healthy condition.

To examine differences in patterns of salivary volatiles among the five molecularly confirmed viral injections, means (by diagnosis) were calculated for the 51 salivary volatiles identified in the final PLS-DA model. Cluster analysis was performed in SAS using PROC CLUSTER using the unweighted pair-group method using arithmetic averages. PROC TREE was used to present clustering results as a dendrogram. Subject age (a predictor in the final PLS-DA model) was not included in this analysis.

**Supplemental References**

Y. Benjamini, Y. Hochberg, Controlling the False Discovery Rate - A Practical and Powerful Approach to Multiple Testing. *Journal of the Royal Statistical Society Series B-Methodological* **57**, 289-300 (1995).

A. Lommen, MetAlign: Interface-Driven, Versatile Metabolomics Tool for Hyphenated Full-Scan Mass Spectrometry Data Preprocessing. Analytical Chemistry 81, 3079-3086 (2009).

Y. M. Tikunov, S. Laptenok, R. D. Hall, A. Bovy, R. C. H. De Vos, MSClust: a tool for unsupervised mass spectra extraction of chromatography-mass spectrometry ion-wise aligned data. Metabolomics 8, 714-718 (2012).

S. Wold, in QSAR: Chemometric Methods in Molecular Design, H. v. d. Waterbeemd, Ed. (Verlag-Chemie, Weinheim, Germany, 1994), pp. 195-218.

Table S1. Post-analysis designation of 11 batches. Dates indicate the first and last day of analyses for a batch, the elapsed number of days, the number of days between batches.

| **Batch** | **Runs** | **First** | **Last** | **Elapsed (Days)** | **Pause (Days)** |
| --- | --- | --- | --- | --- | --- |
| A | 5 | 12-Jan-22 | 26-Jan-22 | 15 | -- |
| B | 2 | 15-Mar-22 | 16-Mar-22 | 2 | 48 |
| C | 2 | 24-May-22 | 26-May-22 | 3 | 69 |
| D | 2 | 6-Oct-22 | 7-Oct-22 | 2 | 133 |
| E | 2 | 16-Nov-22 | 17-Nov-22 | 2 | 40 |
| F | 2 | 6-Dec-22 | 7-Dec-22 | 2 | 19 |
| G | 2 | 10-Jan-23 | 11-Jan-23 | 2 | 34 |
| H | 4 | 14-Mar-23 | 17-Mar-23 | 4 | 62 |
| I | 2 | 21-Jun-23 | 23-Jun-23 | 3 | 96 |
| J | 4 | 28-Nov-23 | 6-Dec-23 | 9 | 158 |
| K | 3 | 20-Feb-24 | 1-Mar-24 | 11 | 76 |
